# Evaluating the association between brain atrophy, hypometabolism, and cognitive decline in Alzheimer’s disease: a PET/MRI study

**DOI:** 10.18632/aging.202580

**Published:** 2021-02-26

**Authors:** Yifan Chen, Junkai Wang, Chunlei Cui, Yusheng Su, Donglai Jing, LiYong Wu, Peipeng Liang, Zhigang Liang

**Affiliations:** 1 Department of Nuclear Medicine, Xuanwu Hospital, Capital Medical University, Beijing, China; 2 Department of Psychology, Tsinghua University, Beijing, China; 3 School of Psychology, Capital Normal University, Beijing, China; 4 Beijing Key Laboratory of Learning and Cognition, Beijing, China; 5 Department of Neurology, Xuanwu Hospital, Capital Medical University, Beijing, China

**Keywords:** hybrid PET/MR, Alzheimer’s disease, hippocampus, default mode network, gray matter volume

## Abstract

Glucose metabolism reduction and brain volume losses are widely reported in Alzheimer’s disease (AD). Considering that neuroimaging changes in the hippocampus and default mode network (DMN) are promising important candidate biomarkers and have been included in the research criteria for the diagnosis of AD, it is hypothesized that atrophy and metabolic changes of the abovementioned regions could be evaluated concurrently to fully explore the neural mechanisms underlying cognitive impairment in AD. Twenty-three AD patients and Twenty-four age-, sex- and education level-matched normal controls underwent a clinical interview, a detailed neuropsychological assessment and a simultaneous 18F-fluoro-2-deoxy-D-glucose positron emission tomography (18F-FDG PET)/high-resolution T1-weighted magnetic resonance imaging (MRI) scan on a hybrid GE SIGNA PET/MR scanner. Brain volume and glucose metabolism were examined in patients and controls to reveal group differences. Multiple linear regression models were employed to explore the relationship between multiple imaging features and cognitive performance in AD. The AD group had significantly reduced volume in the hippocampus and DMN regions (P < 0.001) relative to that of normal controls determined by using ROI analysis. Compared to normal controls, significantly decreased metabolism in the DMN (P < 0.001) was also found in AD patients, which still survived after controlling for gray matter atrophy (P < 0.001). These findings from ROI analysis were further confirmed by whole-brain confirmatory analysis (P < 0.001, FWE-corrected). Finally, multiple linear regression results showed that impairment of multiple cognitive tasks was significantly correlated with the combination of DMN hypometabolism and atrophy in the hippocampus and DMN regions. This study demonstrated that combining functional and structural features can better explain the cognitive decline of AD patients than unimodal FDG or brain volume changes alone. These findings may have important implications for understanding the neural mechanisms of cognitive decline in AD.

## INTRODUCTION

Alzheimer’s disease (AD) is a progressive neurodegenerative disorder with insidious onset followed by cognitive decline [1]. Prior studies have revealed that individuals with mild cognitive impairment (MCI), which represents a transitional stage between normal aging and a very early phase of AD, will face many problems in multiple cognitive domains, including memory, executive function, attention, language, and visuospatial skills [2, 3]. According to the new diagnostic criteria for AD, neuropsychological tests are recognized as fundamental elements of the core clinical criteria [4].

To enhance the pathophysiological specificity of the diagnosis, neuroimaging biomarkers have been incorporated into diagnostic criteria and have been considered important research criteria in the new clinical criteria for AD, MCI due to AD and the preclinical stage of AD, which can be evaluated by high-resolution structural magnetic resonance imaging (MRI) measures of atrophy and fluoro-2-deoxy-D-glucose positron emission tomography (FDG-PET) measures of cerebral hypometabolism [2, 4, 5]. MRI-based measures of brain atrophy are regarded as valid markers of AD and its progression; brain atrophy occurs years before symptoms appear with a stereotypical pattern of early medial temporal lobe (entorhinal cortex and hippocampus) involvement progressively extending to neocortical damage [6, 7]. Consistent with the gradual decline across multiple cognitive domains, atrophy in the hippocampus is associated with behavioral impairment as evaluated by the Mini-Mental State Examination (MMSE) and Auditory Verbal Learning Test (AVLT) [8, 9]. Typical FDG-PET findings in AD have manifested reduced glucose metabolism in the parieto-temporal association cortex, precuneus, and posterior cingulate cortices, which have extensive overlapping regions within the default mode network (DMN) [10–12]. Given that cognitive function depends on neuronal activity in the brain [13], abnormal glucose metabolism observed in AD has been repeatedly reported to be associated with poor cognitive performance [14–16].

Generally, gray matter volume atrophy refers to neuron loss or a reduction in the number of connections between neurons due to apoptosis and injury [6]. Cerebral hypometabolism represents reductions in the cerebral metabolic rate of glucose consumption (CMRglc) [13] and the distribution of synapse dysfunction *in vivo* [17]. Considering that different neuroimaging biomarkers (e.g., brain atrophy and cerebral hypometabolism) reflect distinct pathophysiological aspects of AD, evaluating brain atrophy and hypometabolism data concurrently may improve the present understanding of cognitive decline in AD patients. However, most current studies have focused on the association between single brain area changes (e.g., brain atrophy or glucose metabolic reduction) from single-modality studies and cognitive impairment in AD [8, 14, 15]. Since a single feature from brain atrophy or glucose metabolic reduction is considered one potential factor for AD pathology, such studies could not comprehensively and clearly explain cognitive decline in AD. There are also other studies that have combined multiple features from brain atrophy and hypometabolism to understand the neural correlates of cognitive impairment in AD [18, 19]. Due to FDG-PET imaging and structural imaging data being acquired separately, registration error and information loss in the registration process are inevitable. Thus, the combination data regarding atrophy and metabolic changes obtained by simultaneous FDG-PET and MRI imaging could better clarify the reasons for cognitive impairment in AD patients than the abovementioned methods.

The recently developed hybrid PET/MR scanners combined the sensitivity of PET and the resolution of MR into a single machine, which can simultaneously evaluate brain structure and glucose metabolism and show changes in small anatomical structures more clearly than a single modality [20]. Synchronous scanning can also reduce registration errors and information loss in the registration process, thus providing a representation that is closer to the real situation of brain activity than that achieved when evaluating two modalities by scanning separately [21]. Therefore, PET/MR scanners are the ideal tool to investigate the relationship between multiple imaging features and cognitive performance in AD. To date, published studies on hybrid PET/MR of AD have mainly focused on the relationship between different modalities (e.g., metabolic activity, intrinsic network connectivity and brain volume) [22–25] as well as the relationship between functional image features and cognitive performance [22, 25]. In the present study, by using hybrid PET/MRI, we hypothesized that the combination of atrophy and metabolic changes in the hippocampus and the DMN could be used to comprehensively explore the neural mechanisms underlying cognitive impairment in AD.

## RESULTS

Demographic characteristics and neuropsychological scores are listed in Table 1. There was no significant difference between AD patients and normal controls in mean age, sex distribution or education level (P > 0.05 for all). There were significant differences between the groups in all cognitive domains. Specifically, compared with the NC group, the AD group had worse performance on the AVLT for both immediate and delayed recall, Digit Span Test (Forwards and Backwards), ADL, BNT, CFT, and TMT (P < 0.01 for all).

**Table 1:** Demographic and neuropsychological data between the two groups.

	**AD**	**NC**	**p**
N	23	24	—
Sex (Female/male)	14/9	12/12	0.454
Age (years)	58.74±4.96	54.67±8.69	0.055
Education Level (years)	11.35±3.50	11.67±3.09	0.381
MMSE	14.52±5.23	29.13±1.51	0.000
MoCA	8.70±4.17	27.08±2.84	0.000
AVLT-immediate recall	7.78±4.22	24.42±6.03	0.000
AVLT-delayed recall	0.22±0.52	9.38±2.04	0.000
Digit Span Forwards	6.91±1.28	8±1.14	0.007
Digit Span Backwards	2.35±1.37	5.25±1.22	0.000
ADL	37.22±11.46	20±0	0.000
BNT	15.18±6.12	25.38±3.63	0.000
CDR	1.48±0.63	0.02±0.10	0.000
CFT	6.23±6.49	15.04±1.16	0.000
TMT	150.18±50.36	36.88±44.24	0.000

Note: Group differences in demographic measures were tested using the independent sample t-test and the Chi-square analyses or Fisher exact test for quantitative and qualitative variables. Statistical significance level was set at P < 0.05 (two-tailed). Abbreviations: MMSE, Mini-Mental State Examination; MoCA, Montreal Cognitive Assessment; AVLT, Rey Auditory Verbal Learning Test; ADL, Activities of Daily Living; BNT, Boston Naming Test; CDR, Clinical Dementia Rating; CFT, Rey-Osteirreth Complex Figure Test; TMT, Trail Making Test; AD, Alzheimer’s disease; NC: normal controls.

**Figure 1:**
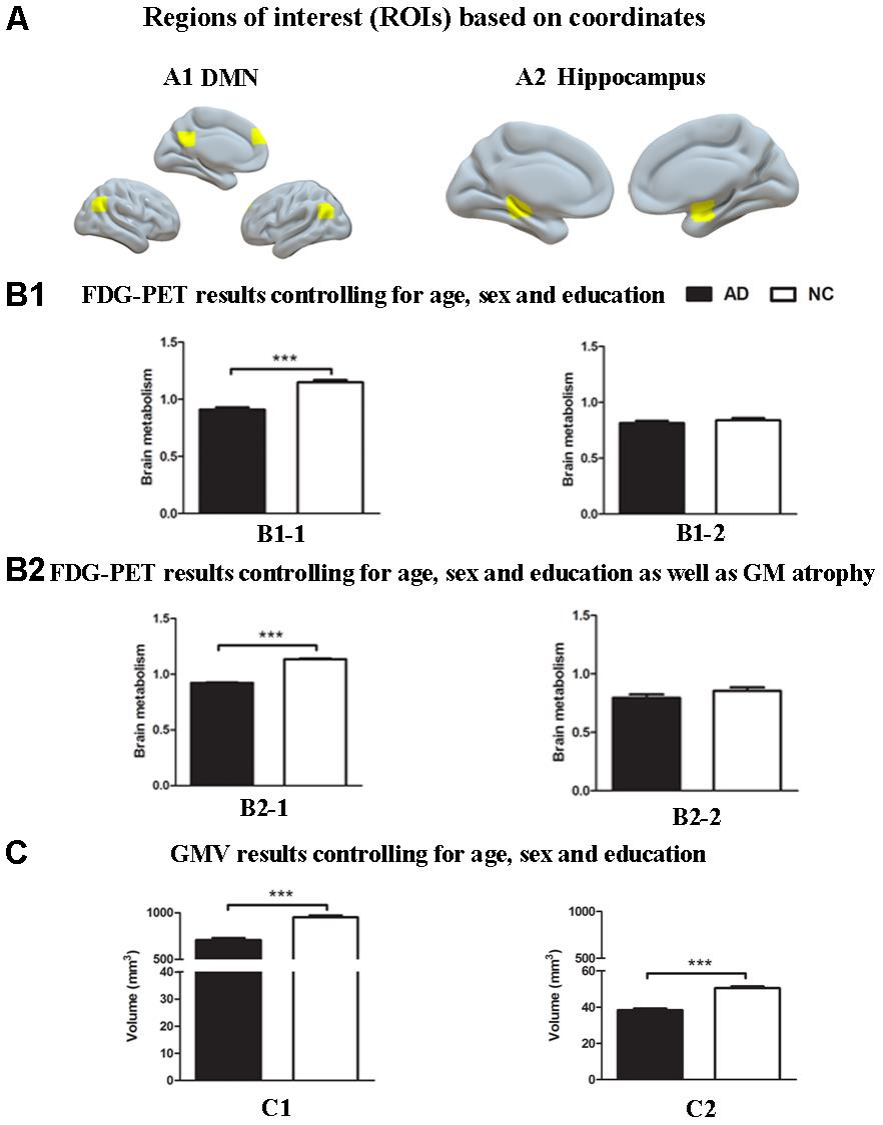
**Group differences in** ^18^**F-FDG SUVR and gray matter volume by using ROI analysis based on coordinates between the AD group and the NC group.** (**A**) ROIs of the DMN (**A1**) and hippocampus (**A2**) were defined based on coordinates (shown in warm yellows). (**B1**) Metabolism results of the DMN (**B1-1**) and hippocampus (**B1-2**) controlling for age, sex and education. (**B2**) Metabolism results of the DMN (**B2-1**) and hippocampus (**B2-2**) controlling for age, sex and education as well as gray matter atrophy. (**C**) Gray matter volume results of the DMN (**C1**) and hippocampus (**C2**) controlling for age, sex and education. Bars represent average metabolism or total gray matter volume and error bars indicate standard error. ***P < .001. Abbreviations: FDG-PET, Fluoro-2-deoxy-D-glucose positron emission tomography; DMN, default mode network; GMV, gray matter volume.

First, ROI analysis based on coordinates was performed to examine the GM volume and brain glucose metabolism differences between the AD group and the NC group. The results showed that the AD group had significantly reduced GM volume in the hippocampus and brain regions within estimated spatial mask of the DMN, including the PCC, the mPFC and bilateral lateral parietal regions, compared with the NC group (P < 0.001, Figure 1C). The AD group also had significantly decreased metabolism within the DMN mask compared to the NC group (P < 0.001, Figure 1B1-1). The reduction in ^18^F-FDG PET metabolism within the DMN mask in the AD group was still significant even after controlling for GM atrophy (P < 0.001, Figure 1B2-1). However, no significant difference was found in brain metabolism within the hippocampus between the two groups (Figure 1B1-2, B2-2).

**Figure 2:**
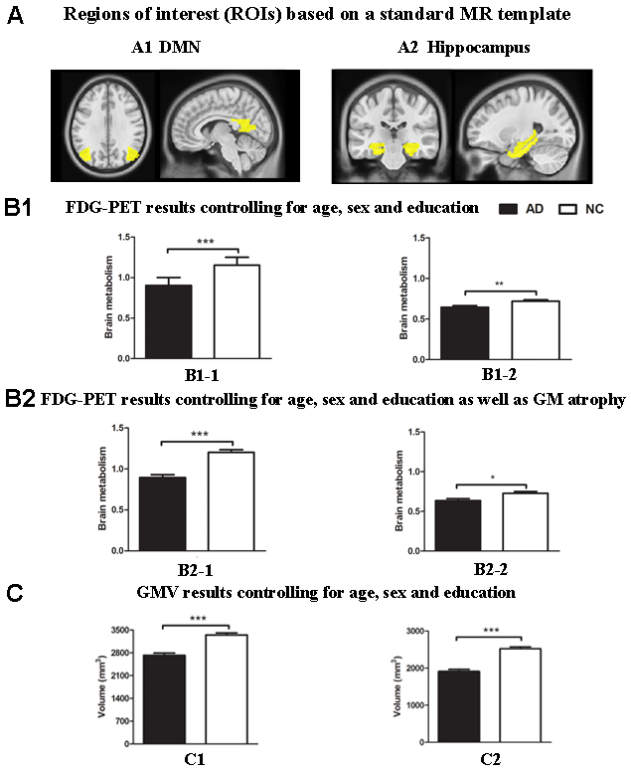
**Group differences in** ^18^**F-FDG SUVR and gray matter volume by using ROI analysis based on template between the AD group and the NC group.** (**A**) ROIs of the DMN (**A1**) and hippocampus (**A2**) were defined based on template (shown in warm yellows). (**B1**) Metabolism results of the DMN (**B1-1**) and hippocampus (**B1-2**) controlling for age, sex and education. (**B2**) Metabolism results of the DMN (**B2-1**) and hippocampus (**B2-2**) controlling for age, sex and education as well as gray matter atrophy. (**C**) Gray matter volume results of the DMN (**C1**) and hippocampus (**C2**) controlling for age, sex and education. Bars represent average metabolism or total gray matter volume and error bars indicate standard error. *P < .05, **P < .01, ***P < .001. Abbreviations: FDG-PET, Fluoro-2-deoxy-D-glucose positron emission tomography; DMN, default mode network; GMV, gray matter volume.

Notably, the results based on the atlas were almost consistent with the findings above. Relative to the NC group, significantly reduced GM volume in the hippocampus and regions within spatial mask of the DMN (P < 0.001, Figure 2C) and decreased metabolism within the DMN mask (P < 0.001, Figure 2B1-1), which survived after controlling for GM atrophy (P < 0.001, Figure 2B2-1), were repeatedly found in the AD group. In addition, the AD group showed significantly decreased ^18^F-FDG PET metabolism within the hippocampus compared to that of the NC group (P < 0.01, Figure 2B1-2, 2B2-2).

**Figure 3:**
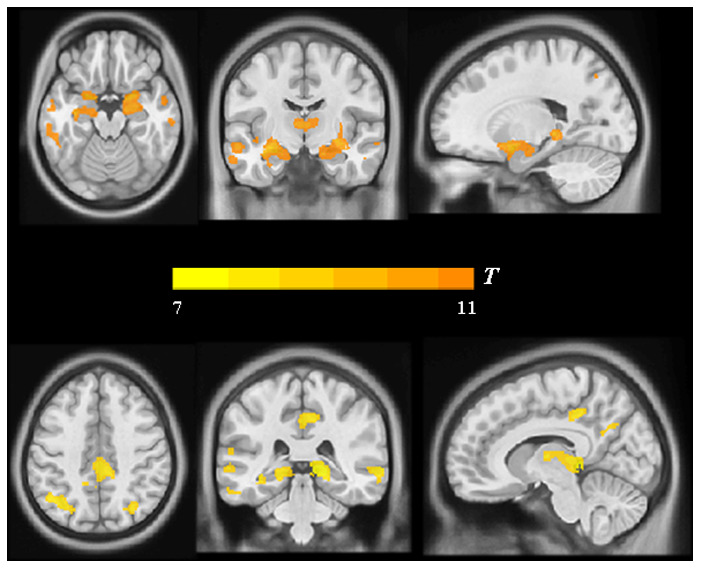
**Group differences in VBM analysis between the AD group and the NC group.** Significant reduced gray matter volume in AD patients was shown in warm yellows.

**Figure 4:**
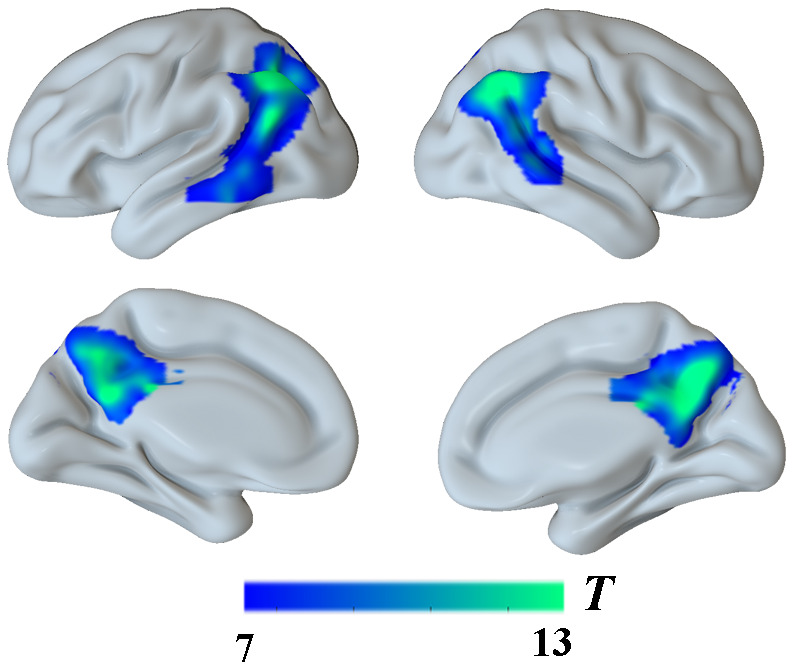
**Group differences in** ^18^**F-FDG SUVR by using whole-brain confirmatory analysis between the AD group and the NC group.** Significant reduced metabolism in AD patients was shown in cold blues.

**Figure 5:**
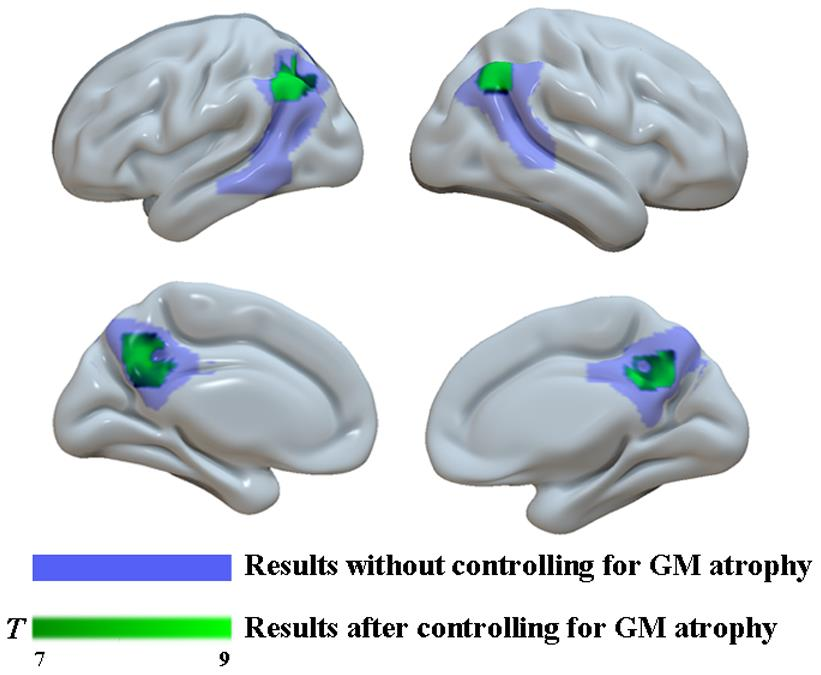
**Group differences in** ^18^**F-FDG SUVR by using whole-brain confirmatory analysis with controlling for GM atrophy between the two groups.** Results without controlling for GM atrophy were shown in blue and results after controlling for GM atrophy were shown in green.

Moreover, the findings described above were further confirmed by whole-brain confirmatory analysis. Relative to the NC group, the results of the AD group were also replicated and showed significantly reduced GM volume in bilateral hippocampus, right amygdala, the PCC and the bilateral angular gyrus (P < 0.001, FWE-corrected, Figure 3 and Table 2), as well as significantly reduced metabolism in multiple regions including the PCC and bilateral angular gyrus within the DMN (Figure 4 and Table 2), which was still significant after regressing out the whole-brain GM volume (Figure 5 and Table 2).

**Table 2:** Group difference in brain metabolism and grey matter volume by using whole-brain confirmatory analysis (FWE corrected for multiple comparisons across the entire volume).

**ROI**	**Brain regions**	**Cluster size**	**T value**	**Peak MNI coordinates**		
				**X**	**Y**	**Z**
**FDG-PET**						
**AD < NC**						
**DMN**						
	L Angular	1596	13.12	-42	-62	50
	R Angular	1339	12.07	52	-62	46
	PCC	2034	7.72	0	-54	36
**DMN** **(GMV-controlled)**						
	L Angular	95	8.72	-42	-62	50
	R Angular	57	7.79	50	-60	42
	PCC	168	7.73	0	-52	36
**VBM**						
**AD < NC**						
	R PHG	471	10.93	11	-36	-2
	R Amygdala	210	10.01	21	0	-14
	L PHG	373	9.17	-15	0	-17
	R MTG	264	9.15	57	-55	19
	L MTG	189	9.07	-54	-18	-11
	PCC	154	8.17	9	-35	42
	L Angular	136	9.01	-35	-64	45
	R Angular	67	7.92	33	-66	45

Voxel level P < 0.001 after FWE correction. Abbreviations: PCC, posterior cingulate cortex; PHG, parahippocampal gyrus; MTG, middle temporal gyrus; FDG-PET, Fluoro-2-deoxy-D-glucose positron emission tomography; DMN, default mode network; GMV, gray matter volume; VBM, voxel-based morphometry; AD, Alzheimer’s disease; NC: normal controls.

Finally, multiple linear regression was used to explore the relationship between multiple imaging features and cognitive performance in the two groups. Metabolism within the hippocampus was not included in the regression model because no significant difference was found in hippocampal metabolism between the two groups. Thus, brain metabolism within the DMN mask, the GM volume of the hippocampus and the GM volume of regions within the DMN mask were chosen as independent variables, and various cognitive test results were considered dependent variables. Control variables were age, sex, and global GM volume. All three variables significantly explained the variance in MMSE scores (F3, 18 = 6.60, p = 0.003) with r2 = 0.52, AVLT scores (F3, 18 = 5.23, p = 0.009) with r2 = 0.47, ADL scores (F3, 18 = 4.98, p = 0.011) with r2 = 0.45 and TMT scores (F3, 18 = 4.43, p = 0.018) with r2 = 0.44. Among the individual explanatory variables, GM volume of regions within the DMN mask was significantly associated with MMSE scores (standardized β = 0.82, p = 0.001), AVLT scores (standardized β = 0.71, p = 0.005), ADL scores (standardized β = 0.68, p = 0.007) and TMT scores (standardized β = 0.80, p = 0.003). GM volume of the hippocampus was significantly associated with AVLT scores (standardized β = 0.31, p = 0.045) and ADL scores (standardized β = -0.33, p = 0.037) (Table 3). Moreover, no significant association was found between multiple imaging features and cognitive performance in the NC group.

**Table 3:** The result of multiple linear regression analyses.

**Dependent variable**	**R**	**R**^2^	**Significant**	**Predictor**	**std.β**	***P***
MMSE	0.724	0.524	**0.003**	FDG_DMN	-0.299	0.085
				Volume_DMN	0.819	**< 0.001**
				Volume_Hip	0.213	0.105
MoCA	0.578	0.335	0.057	FDG_DMN	-0.372	0.075
				Volume_DMN	0.720	**0.005**
				Volume_Hip	0.083	0.337
AVLT	0.682	0.466	**0.009**	FDG_DMN	-0.258	0.130
				Volume_DMN	0.707	**0.003**
				Volume_Hip	0.311	**0.045**
ADL	0.673	0.454	**0.011**	FDG_DMN	0.240	0.149
				Volume_DMN	-0.676	**0.004**
				Volume_Hip	-0.332	**0.037**
TMT	0.662	0.439	**0.018**	FDG_DMN	-0.264	0.134
				Volume_DMN	0.795	**0.002**
				Volume_Hip	-0.082	0.330

Abbreviations: MMSE, Mini-Mental State Examination; MoCA, Montreal Cognitive Assessment; AVLT, Rey Auditory Verbal Learning Test; ADL, Activities of Daily Living; TMT, Trail Making Test; FDG_DMN, brain metabolism within the DMN; Volume_DMN, volume of the DMN; Volume_Hip, volume of hippocampus; FDG, Fluoro-2-deoxy-D-glucose; DMN, default mode network; Hip, hippocampus.

In addition, contrary to the multiple linear regression findings, partial Pearson’s correlation analysis was also performed to test the association between a single parameter (e.g., brain atrophy or glucose metabolic reduction) and cognitive performance in the AD group. No correlation was found between brain metabolism within the DMN mask and cognitive variables or between GM volume of the hippocampus, GM volume of the regions within the DMN mask and cognitive variables after p-value correction for multiple comparisons (corrected P > 0.05 for all, Supplementary Table 1).

## DISCUSSION

The present study comprehensively explored the neural mechanisms underlying cognitive impairment in AD. Hypometabolic DMN and GM atrophy of the hippocampus and DMN regions was repeatedly detected in the AD group. Significant relationships between the above variables and multiple cognitive tests were also observed in AD patients. These findings may deepen our understanding of neural correlates of cognitive impairment in AD.

In line with the previous literature [11, 12, 26], significant GM atrophy and a reduction in metabolism within the DMN were detected in AD patients even when we set a strict threshold for whole-brain analysis (P < 0.001, FWE-corrected). GM volume had significant correlation with the glucose metabolism and both measures could accurately differentiate the AD group from the HC group (Supplementary Figure 1 and Supplementary Table 2). In general, during early clinical stages of AD, patterns of brain atrophy and glucose hypometabolism converge across wide regions of the DMN, supporting the central role of the DMN in AD [27]. It is worth noting that inconsistent results of hippocampal glucose metabolism were found by applying three different analytical methods in this study, and the AD group had slight hippocampal hypometabolism detected only by using atlas-based analysis. According to prior studies, both reduced hippocampal metabolism and preserved hippocampal metabolism have been reported in AD patients [24, 28, 29]. One possible explanation is that there are discrepancies depending on the age of disease onset. In presenile-onset AD, the reduction in glucose metabolism is often centered in the parietotemporal association cortex, which is consistent with our study sample, whereas in senile-onset AD, glucose metabolism tends to be reduced in the limbic system and in the frontal lobe, which would be in line with studies reporting reduced hippocampal metabolism [30, 31]. The other possible explanation is the heterogeneity of hippocampal subregion metabolism in AD patients, which showed lower glucose metabolism in specified subregions [22]. Due to selection bias, our selected coordinates may be located in subregions that had preserved hippocampal metabolism. For this reason, coordinate-based analysis did not detect reduced hippocampal metabolism in AD patients, whereas the AD group exhibited slight hippocampal hypometabolism based on atlas-based analysis. Considering presenile-onset AD in the current study and a strict threshold for whole-brain analysis, reduced hippocampal metabolism was not found in AD patients by using whole-brain confirmatory analysis.

Global cognitive function in AD patients can be well explained by the model including all three parameters (52.4% variance of MMSE). The volume of the DMN significantly contributes to regression, and glucose metabolism in the DMN shows a trend toward significance. The DMN has been found to be involved in various domains of cognitive processing, including episodic memory, visuospatial imagery, attention, self-referential processing, language, etc. [32, 33]. Atrophy is accompanied by damaged synapse and metabolite changes [6]. Due to neuron losses and disrupted brain activity in the DMN regions, atrophy may cause dysfunction of the DMN, which is considered a hallmark of AD [34]. In addition, the multimodel also explained 33.5% of the variance in the MoCA results in AD patients, which is a lower amount of explainable variance than that for the MMSE results. This may be attributed to the floor effect [35]. Several studies have confirmed that the MoCA is a more suitable screening tool for patients with MCI whose disease is prodromal AD [36, 37].

As a global cognitive screening measure, performance on the ADL scale was significantly explained by three variables. Both the volume of the DMN and hippocampus significantly contribute to regression. For daily living ability, several cognitive domains, including episodic memory, speed of processing and verbal ability, in which the DMN and hippocampus are involved, are critical to maintain the normal level of daily life activities [38, 39]. Previous studies also revealed that global cognitive function evaluated by the MMSE was related to ADL performance [40, 41]. From this point of view, it is understandable that the multimodel can explain 45.4% of the variance in ADL scores in AD patients.

It is common knowledge that the hippocampus is most consistently associated with episodic memory. Based on several recent studies, certain areas of the parietal cortex that are commonly understood to be part of the DMN are involved in episodic memory and, together with the hippocampus, constitute a hippocampal-parietal network supporting memory function [42–45]. In line with this, performance on the AVLT in AD patients was well explained by the multimodel (46.6% variance of AVLT scores), and the volume of both of the DMN and hippocampus significantly contributed to regression.

Additionally, the combination of functional and structural parameters also associated with performance on the TMT and the volume of the DMN significantly contributes to regression. As a high-level cognitive ability, executive cognitive function is considered to involve multiple domains, such as planning, goal management, cognitive flexibility, inhibition, and judgment [46]. In accordance with complex functions, distributed brain regions that include the prefrontal cortex, the parietal cortex, the posterior cingulate gyrus, the insula, and the temporal cortex [47] are critical substrates for executive processes. Therefore, a loss of neurons and the connections between neurons in the DMN, which contains key nodes of executive-related regions, results in a disruption of the integrity of the neural circuitry underlying executive function and poor TMT performance in AD patients.

Some limitations of this study need to be addressed. First, we only focused on the volume of the brain structure. In the next research phase, we need to further investigate the mechanism of cognitive impairment by combining various morphological parameters, such as cortical thickness and surface area. Second, in the present study, we only focused on the changes in FDG-PET in the resting state. However, the cognitive process is a dynamic process, and further research on the dynamic FDG-PET changes in the cognitive task state is needed to comprehensively reveal the mechanism of cognitive impairment. Third, the sample size in the current study is relatively small. However, our neuroimaging results are in line with the previous literature, and a strict threshold for whole-brain analysis is employed to examine differences between AD patients and normal controls. We are reasonably confident about the observed significant results. Future studies should increase the sample size to confirm the relationship between functional image features and cognitive performance. Fourth, multiple tracers, such as tau PET and Aβ PET, are needed to observe the pathological changes in the whole course of the disease to confirm the initial cause of cognitive impairment. Finally, the diagnosis of AD is made on the basis of clinical examinations rather than on a pathological basis.

## CONCLUSIONS

In summary, hypometabolism in the DMN and brain atrophy of the hippocampus and DMN-related regions were repeatedly detected in AD patients by using different analytical methods. Based on the findings above, data obtained using a combination of functional and structural neuroimaging features can better explain the cognitive decline of AD patients than data obtained using unimodal FDG or volume alone. These findings may have important implications for understanding the neural mechanisms of cognitive decline in AD.

## MATERIALS AND METHODS

### Subjects

Twenty-three AD patients (mean age 58.74 ± 4.96 years) and twenty-four normal controls (NCs; mean age 54.67 ± 8.69 years) participated in the study. All AD patients were recruited from the Memory Clinic of the Department of Neurology at XuanWu Hospital, Capital Medical University. Normal controls were recruited from the local community by advertisements. All participants received financial compensation for their participation. The study protocol was approved by the Institutional Review Board of XuanWu Hospital at Capital Medical University, and written informed consent was obtained from all participants or their legal relatives after the study protocol had been fully explained.

All participants underwent a standardized assessment protocol that included medical history, neurological and psychiatric examination, a battery of neuropsychological tests and an ^18^F-FDG PET/MR examination. All patients were diagnosed with AD according to the Diagnostic and Statistical Manual of Mental Disorders-V (DSM-V) criteria for Alzheimer's dementia [48] and the National Institutes on Aging and Alzheimer’s Association (NIA-AA) [4]. Individuals with no cognitive complaints and normal performance on the standardized neuropsychological tests were included as normal controls. The following exclusion criteria were applied to all participants: 1) presenting with any serious medical, psychiatric, or neurological disorders that could affect cognitive function (e.g., substance abuse, alcoholism, schizophrenia, brain tumors, or cerebrovascular disease); 2) standard contraindications for MR imaging examinations (such as magnetic metal implants or pacemakers); 3) evidence of focal brain lesions on MRI (e.g., stroke lesions or bleeding); 4) the presence of severe behavioral or communication problems that would make a clinical MRI examination incomplete; and 5) the absence of a reliable informant.

### Neuropsychological assessments

The neuropsychological test battery consists of widely used neuropsychological assessments measuring cognitive function in the domains of memory, language, and executive function. Global cognitive screening measures included the MMSE [49], the Montreal Cognitive Assessment (MoCA) [50], the Clinical Dementia Rating (CDR) scale [51] and the Activities of Daily Living (ADL) scale [40]. Word list memory was evaluated with Rey’s Auditory-Verbal Learning Test (AVLT) [52]. Working memory was measured with the Digit Span Forwards and Backwards test from the Wechsler Adult Intelligence Scale-III [53]. Executive function was evaluated with the Trail Making Test (TMT) [54]. Language was measured with the Boston Naming Test (BNT) [55]. Visuo-construction abilities were assessed by the Rey-Osterrieth Complex Figure Test (CFT) [56]. One AD patient failed to perform the required cognitive tasks, including the BNT, TMT and CFT, due to a lack of understanding regarding task execution.

### PET/MR acquisition protocol

All images were acquired on a hybrid 3.0 T TOF PET/MR (SIGNA PET/MR, GE Healthcare, WI, USA) [57]. PET and MR images were simultaneously acquired in a vendor-supplied 19-channel head and neck union coil. 3D BRAVO T1-weighted sagittal images and FDG-PET volumes were acquired in the same session. Additionally, a FLAIR sequence was acquired to screen for brain lesions and abnormalities.

The data was acquired with protocols in line with the procedure guidelines for PET brain imaging provided by the European Association of Nuclear Medicine (EANM) [58]. Every subject was asked to fast for at least 6 h to reach a serum glucose level lower than 9 mmol/l and received an intravenous injection of ^18^F-FDG (3.7 MBq/kg) [59]. Participants were positioned in a quiet, warm and dimly lit room at least 30 minutes before FDG administration and during the brain uptake phase. Then, they were placed in a hybrid PET/MR scanner as were made as comfortable as possible, with head restraints to minimize motion artifacts. The imaging parameters are described below.

3D BRAVO: repetition time (TR) = 6.9 ms, echo time (TE) = 2.98 ms, flip angle = 12° C, inversion time (TI) = 450 ms, matrix size = 256 × 256, field of view = 256×256 mm2, slice thickness = 1 mm, 192 sagittal slices with no gap, voxel size = 1×1×1 mm3, and acquisition time = 4 minutes 48 seconds.

PET: Static FDG-PET data were acquired in list mode for 30 minutes and comprised 89 slices covering the whole brain. Matrix size = 192×192, field of view = 350×350 mm2, pixel size = 1.82×1.82×2.78 mm3, including corrections for random coincidences, dead time, scatter and photon attenuation. Attenuation correction was performed based on MR imaging of the brain (Atlas-based coregistration of 2-point Dixon) [59], and the default attenuation correction sequence was automatically prescribed and acquired as follows: LAVA-Flex (GE Healthcare) axial acquisition, TR = 4 ms, TE = 1.7 ms, slice thickness = 5.2 mm with 2.6 mm overlap, 120 slices, pixel size = 1.95 × 2.93 mm, and acquisition time = 18 seconds. The images were reconstructed with a time-of-flight point spread function and the order subset-expectation maximization (TOF-PSF-OSEM) algorithm (32 subsets, 8 iterations and a 3-mm cutoff filter) [21].

### Data processing

The preprocessing of PET and MRI data is described in detail as follows. Briefly, PET and T1 images were first checked for visible quality issues, and one patient was excluded due to severe motion artifacts. Then, the static PET images were preprocessed by using statistical parametric mapping (SPM12; http://www.fil.ion.ucl.ac.uk/spm/software/spm12) implemented in MATLAB (MathWorks, Natick, Massachusetts). The structural MRI images were normalized to standard Montreal Neurological Institute (MNI) space using diffeomorphic anatomical registration through exponentiated lie algebra (DARTEL) normalization as implemented in SPM12. After normalization, the transformation parameters determined by T1-weighted image spatial normalization were then applied to the coregistered PET images for PET spatial normalization. The images were then smoothed using an isotropic Gaussian kernel with a full width at half maximum of 8 mm for all directions. Finally, PET scan intensity was normalized using a whole cerebellum reference region to create standardized uptake value ratio (SUVR) images. Since FDG-PET signals arise mainly from gray matter, the partial volume effect from nongray matter may potentially influence the results. Therefore, the group comparison was assessed only for gray matter (GM) voxels selected by applying a threshold to the GM probability maps, and we also applied a strict threshold (P < 0.001, FWE-corrected) to acquire very reliable results.

The T1-weighted 3D BRAVO images were processed using the voxel-based morphometry (VBM) toolbox based on SPM12. Briefly, MR images were segmented into GM, white matter (WM) and cerebrospinal fluid (CSF) partitions. Subsequently, the GM and WM partitions of each subject in native space were high dimensionally registered and normalized to the standard MNI space using diffeomorphic anatomical registration through exponentiated lie algebra (DARTEL) normalization as implemented in SPM12. This improved method can achieve more accurate intersubject coregistration of brain images. After normalization, the images with modulation were smoothed with a Gaussian filter of an 8 mm full-width half-maximum kernel.

### Data analysis

ROI-based analysis of structural MRI and PET images was performed in the current study. First, multiple brain regions based on a priori coordinates within the DMN and the hippocampus were employed to investigate group differences. To ensure repeatability and reliability of the results in the current study, the DMN and the hippocampus were considered as a whole to define ROIs. Multiple coordinates in the DMN and the hippocampus were defined from the literature [60, 61]. A diameter of 4 mm was used for hippocampal ROIs, and a diameter of 6 mm was used for ROIs in the DMN. ROIs in the DMN included regions centered in the posterior cingulate cortex (PCC: MNI coordinates: 0, -52, 27), the medial prefrontal cortex (mPFC: MNI coordinates: -1, 54, 27), the left lateral parietal cortex (LLP: MNI coordinates: -46, -66, 30), and the right lateral parietal cortex (RLP: MNI coordinates: 49, -63, 33). Hippocampal ROIs were located in the left and right hippocampus (MNI coordinates: -24, -30, -6/21, -6, -18). Then, to avoid coordinate-based selection bias, atlas-based analysis was performed to validate the results acquired by ROI analysis based on coordinates. The masks of the DMN and hippocampus were generated from neuroanatomic and cytoarchitectonic atlases by using WFU PickAtlas [62].

Two sets of ROI signals were extracted from the SUVR on ^18^F-FDG SUVR images and smoothed T1 images. Analysis of covariance (ANCOVA) was used to test the differences between the AD group and the NC group in the mean brain metabolism and total gray matter volume within the DMN and hippocampus with age, sex and years of education as covariates. To examine the effect of gray matter atrophy on brain glucose metabolism, we also performed an extra ANCOVA test on FDG-PET images with age, sex, years of education and global gray matter volume as covariates. A value of P < 0.05 was considered statistically significant.

Moreover, to avoid missing any other brain atrophy and hypometabolism data in the AD group, the processed FDG-PET and structural images were further used to perform whole-brain confirmatory analysis between the AD group and the NC group using a two-tailed two-sample t-test with the aforementioned variables as covariates. A voxel level threshold was set at P < 0.001 (FWE-corrected).

### Statistical analysis

SPSS (version 21.0, IBM) was utilized for statistical analyses. Group differences in demographic measures were tested using the independent sample t-test and the chi-square analyses or Fisher’s exact tests were used for quantitative and qualitative variables. To compare cognitive variables, analysis of covariance (ANCOVA) was conducted with age, sex, and years of education as covariates. A P-value < 0.05 was considered statistically significant. In the final step of analysis, we tested whether functional and structural impairments within the DMN and hippocampus were associated with cognitive deficits in the AD group. Partial Pearson’s correlations between gray matter volume within ROIs, FDG SUVR within ROIs, and clinical assessments were first calculated in the AD group. Then, we performed multiple linear regression analysis using SPSS, with MMSE, MoCA, AVLT, ADL, BNT, CFT and TMT results as dependent variables, and the independent variables were ^18^F-FDG PET metabolism within the DMN, volume of the DMN regions and volume of the hippocampus. Additional control variables of no interest were age, sex, years of education and global gray matter volume.

## Supplementary Material

Supplementary Figure 1

Supplementary Tables
