# Supplementary material for: Evaluating the association between brain atrophy, hypometabolism, and cognitive decline in Alzheimer’s disease: a PET/MRI study
Source: Aging (Albany NY). 2021 Feb 26;13(5):7228–46. doi: 10.18632/aging.202580 (PMC7993730; doi:10.18632/aging.202580)
Supplement: Supplementary Figure 1 [file aging-13-202580-s001.pdf]

SUPPLEMENTARY FIGURE

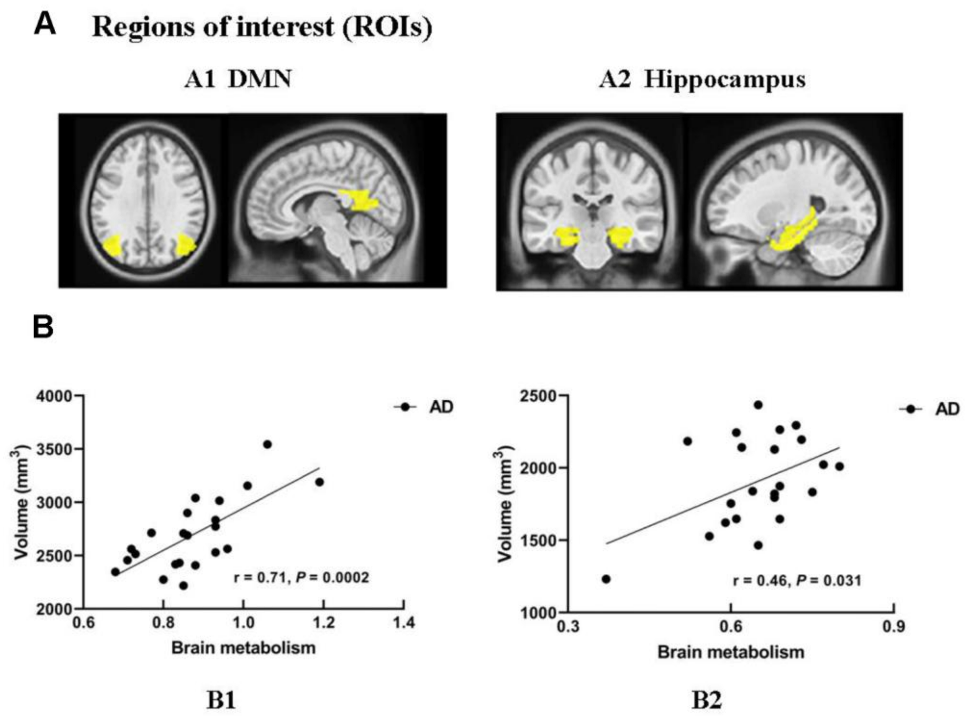

Supplementary Figure 1. The relationship between SUVR and brain volumes in the AD group. (**A**) ROIs of the DMN (**A1**) and hippocampus (**A2**) were defined based on template (shown in warm yellows). (**B1**) The GM volume of regions within the DMN had correlation with the 18F-FDG SUVR within the DMN mask. (**B2**) The GM volume in the hippocampus showed correlation with the 18F-FDG SUVR of hippocampus. Abbreviations: FDG, Fluoro-2-deoxy-D-glucose; DMN, default mode network.
