# Supplementary material for: Evaluating the association between brain atrophy, hypometabolism, and cognitive decline in Alzheimer’s disease: a PET/MRI study
Source: Aging (Albany NY). 2021 Feb 26;13(5):7228–46. doi: 10.18632/aging.202580 (PMC7993730; doi:10.18632/aging.202580)
Supplement: Supplementary Tables [file aging-13-202580-s002.pdf]

SUPPLEMENTARY TABLES

Supplementary Table 1. Brain-behavior correlations in AD subjects.

Neuroimaging measures	Behavioral measures	Correlation coefficient	*P* value
FDG_DMN	MMSE	0.027	0.915
	MoCA	0.032	0.901
	AVLT	-0.029	0.910
	DST	0.044	0.863
	ADL	-0.085	0.736
	BNT	0.276	0.267
	CFT	0.365	0.136
	TMT	0.157	0.535
Volume_DMN	MMSE	0.467	0.059
	MoCA	0.272	0.290
	AVLT	0.245	0.344
	DST	-0.158	0.544
	ADL	-0.235	0.364
	BNT	-0.295	0.251
	CFT	0.278	0.279
	TMT	0.559	0.020
Volume_Hip	MMSE	0.128	0.612
	MoCA	0.040	0.876
	AVLT	0.251	0.315
	DST	0.350	0.154
	ADL	-0.309	0.212
	BNT	0.277	0.267
	CFT	0.067	0.791
	TMT	-0.152	0.547

Note: Partial Pearson's correlation analysis was also performed to test the association between single parameter (e.g. brain atrophy or glucose metabolic reduction) and cognitive performance in the AD group. Statistical significance level was set at corrected $P < 0.05$ (two-tailed). Abbreviations: AD, Alzheimer's disease; MMSE, Mini-Mental State Examination; MoCA, Montreal Cognitive Assessment; AVLT, Rey Auditory Verbal Learning Test; DST, Digit Span Test; ADL, Activities of Daily Living; BNT, Boston Naming Test; CFT, Rey-Osteirreth Complex Figure Test; TMT, Trail Making Test; NC, normal controls; FDG_DMN, brain metabolism within the DMN; Volume_DMN, volume of the DMN; Volume_Hip , volume of hippocampus; FDG, Fluoro-2-deoxy-D-glucose; DMN, default mode network; Hip, hippocampus.

Supplementary Table 2. Summary of logistic regression analysis for different MRI measure used as a predictor of AD.

(A1) Brain metabolism as outcome predictors.

Predictor	B	SE B	Wald	*P* value
Brain metabolism within the DMN mask	28.35	10.30	7.57	**0.006**

(A2) GM volume as outcome predictors.

Predictor	B	SE B	Wald	*P* value
GM volume of the hippocampus	0.020	0.008	6.66	**0.010**

(B1) The classification table of a model containing brain metabolism within the DMN mask.

Observed outcome	Predicted outcome		Percentage correct
	AD	NC	
AD	21	1	95.5
NC	0	24	100.0
Overall accuracy percentage of index			97.8

Note: Model coded 0 for AD and 1 for NC. Abbreviations: AD, Alzheimer's disease; NC, normal controls; B, raw Beta coefficient; SE B, standard error for raw Beta coefficient.

(B2) The classification table of a model containing GM volume of the hippocampus.

Observed outcome	Predicted outcome		Percentage correct
	AD	NC	
AD	21	1	95.5
NC	2	22	91.7
Overall accuracy percentage of index			93.5

Note: Model coded 0 for AD and 1 for NC. Abbreviations: AD, Alzheimer's disease; NC, normal controls; B, raw Beta coefficient; SE B, standard error for raw Beta coefficient.
